# ICTV Virus Taxonomy Profile: *Retroviridae* 2021

**DOI:** 10.1099/jgv.0.001712

**Published:** 2021-12-23

**Authors:** John Coffin, Jonas Blomberg, Hung Fan, Robert Gifford, Theodora Hatziioannou, Dirk Lindemann, Jens Mayer, Jonathan Stoye, Michael Tristem, Welkin Johnson, ICTV Report Consortium

**Affiliations:** ^1^​ Tufts University, Boston, MA 2111, USA; ^2^​ Uppsala University, Sweden; ^3^​ University of California, Irvine, CA 92697-3905, USA; ^4^​ Center for Virus Research, Glasgow G61 1QH, UK; ^5^​ The Rockefeller University, New York, NY10065, USA; ^6^​ Technische Universität Dresden, Dresden, 01307, Germany; ^7^​ University of Saarland, 66421 Homburg/Saar, Germany; ^8^​ Francis Crick Institute, 1 Midland Road, London NW1 1AT, UK; ^9^​ Imperial College London, Berkshire, SL5 7PY, UK; ^10^​ Boston College, Chestnut Hill, MA 02467, USA

**Keywords:** ICTV Report, taxonomy, *Retroviridae*, HIV, AIDS

## Abstract

Viruses in the family *Retroviridae* are found in a wide variety of vertebrate hosts. Enveloped virions are 80–100 nm in diameter with an inner core containing the viral genome and replicative enzymes. Core morphology is often characteristic for viruses within the same genus. Replication involves reverse transcription and integration into host cell DNA, resulting in a provirus. Integration into germline cells can result in a heritable provirus known as an endogenous retrovirus. This is a summary of the International Committee on Taxonomy of Viruses (ICTV) Report on the family *Retroviridae*, which is available at ictv.global/report/retroviridae.

## Abbreviations

LTR, long terminal repeat; SU, Env surface subunit; TM, Env transmembrane subunit.

## Virion

**Fig. 1.**
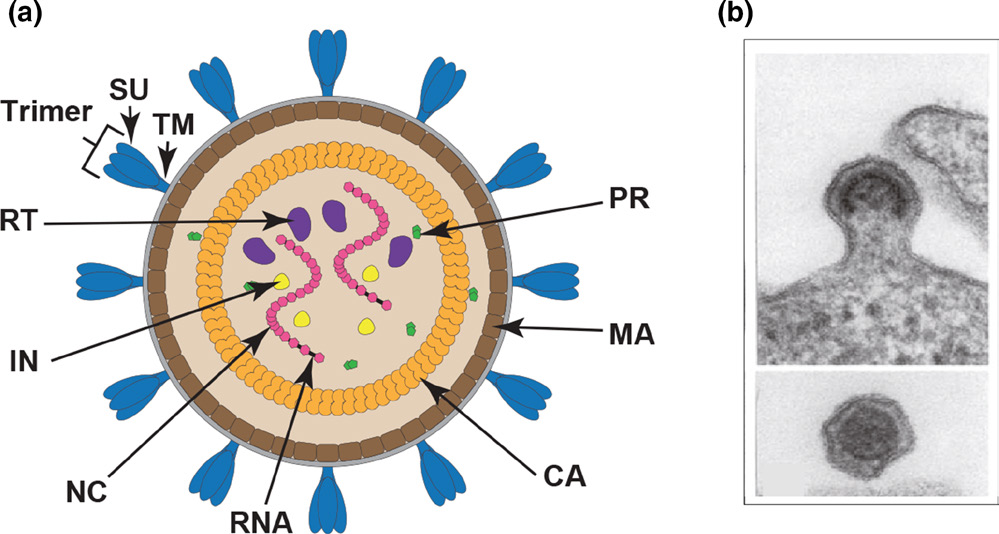
(**a**) Schematic diagram of a retrovirus particle (courtesy of B. Lawhorn). SU, surface and TM, transmembrane subunits of the envelope protein; RT, reverse transcriptase; IN, integrase; NC, nucleocapsid; CA, capsid; MA, matrix; PR, protease. (**b**) Transmission electron micrograph of a murine leukemia virus particle. Credit M. Gonda, reproduced from [4], by permission of Cold Spring Harbor Laboratory Press.

Virions are spherical, enveloped and 80–100 nm in diameter (Table 1, Fig. 1) [1] with 8 nm-long glycoprotein surface projections. The internal core constitutes the viral nucleocapsid. The apparently spherical nucleocapsid is rod or truncated cone-shaped for members of the genus *Lentivirus,* eccentric for members of the genus *Betaretrovirus* and concentric for members of the genera *Alpharetrovirus*, *Gammaretrovirus*, *Deltaretroviru*s and the subfamily *Spumaretrovirinae*.

**Table 1.** Characteristics of members of the family *Retroviridae*

Example:	Moloney murine leukemia virus (AF033811), species *Murine leukemia virus*, genus *Gammaretrovirus*
Virion	Enveloped spheres of 80–100 nm diameter with 8 nm glycoprotein spikes
Genome	Dimer of positive-sense, ssRNA (7–13 kb); may be partially reverse-transcribed in virions of spumaretroviruses
Replication	dsDNA produced by reverse transcription of the RNA genome is integrated into host genome and serves as template for transcription
Translation	From capped and polyadenylated genomic transcripts and subgenomic, spliced mRNAs
Host range	Vertebrates
Taxonomy	Realm *Riboviria*, kingdom *Pararnavirae*, phylum *Artverviricota*, class *Revtraviricetes*, order *Ortervirales*; the subfamilies *Orthoretrovirinae* and *Spumaretrovirinae* include >10 genera and >65 species

## Genome

The genome of members of the subfamily *Orthoretrovirinae* is a dimer of linear positive-sense ssRNA of 7–13 kb [1]. The component monomers are linked by hydrogen bonds, and are polyadenylated at the 3′-end and possess a type 1 5′-cap structure. Purified virion RNA is not infectious. Each monomer is associated with a specific tRNA molecule that is base-paired to the primer binding site near the 5′-end of the RNA. A proportion of virions of members of the *Spumaretrovirinae* may contain dsDNA (5–10 %), derived from reverse transcription of viral genomic RNA genome during assembly and egress from the cell.

## Replication

**Fig. 2.**
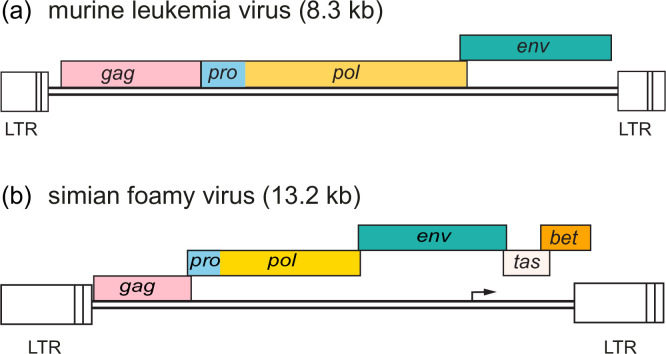
Genome organization of representatives of the subfamilies (a) *Orthoretrovirinae* and (b) *Spumaretrovirinae*. Arrowhead indicates an internal promoter found in the foamy virus genome.

The entry glycoprotein Env forms a heterotrimer comprising surface (SU) and transmembrane (TM) subunits. Binding is mediated by the SU subunit to specific receptors on the cell surface, resulting in fusion of the viral envelope with the plasma membrane [2, 3]. Reverse transcription of the RNA genome produces dsDNA with long terminal repeats (LTRs) (Fig. 2) [4]. The dsDNA is integrated into the host cell genome to form the provirus [5], which serves as a template for synthesis of viral genomes and mRNAs by RNA polymerase II. Capsids assemble either at the plasma membrane (members of most genera) or as intracytoplasmic particles (for members of the subfamily *Spumaretrovirinae* and members of the genus *Betaretrovirus*). Virions are released from the cell by budding and undergo proteolytic maturation [6].

## Pathogenicity

Many retroviruses are important human and veterinary pathogens, associated with a variety of diseases, including leukemias, lymphomas, sarcomas and other tumours of mesodermal origin; carcinomas of mammary tissue, liver, lung and kidney; immunodeficiencies (e.g. AIDS); autoimmune diseases; motor neuron diseases; and several acute diseases involving tissue damage [4, 7]. Transmission is horizontal via a number of routes, including blood, saliva and sexual contact, and via direct infection of the developing embryo, or via milk or perinatal routes.

## Taxonomy

Current taxonomy: ictv.global/taxonomy. The family *Retroviridae* belongs to the order *Ortervirales* [8]. Currently, classification of subfamilies, genera and species is based primarily on phylogenetic analysis, genome characteristics (ssRNA or ssRNA partially reverse-transcribed in the virion, size) and the presence or absence of specific regulatory and accessory genes.

## Resources

Full ICTV Report on the family *Retroviridae*: ictv.global/report/retroviridae.
